# Identification of *ARF* family in blueberry and its potential involvement of fruit development and pH stress response

**DOI:** 10.1186/s12864-022-08556-y

**Published:** 2022-04-27

**Authors:** Xuyan Li, Xiaoyi Zhang, Tianran Shi, Min Chen, Chengguo Jia, Jingying Wang, Zhixia Hou, Junyou Han, Shaomin Bian

**Affiliations:** 1 College of Plant Science, Jilin University, Changchun, China; 2 Key Laboratory for Silviculture and Conservation of Ministry of Education, Beijing Forestry University, Research & Development Center of Blueberry, Beijing, 100083 China

**Keywords:** Blueberry, *ARF* family, Gene structure, Domain and motif compositions, Expression pattern, Fruit development, Response to pH stress

## Abstract

**Background:**

Auxin responsive factor (ARF) family is one of core components in auxin signalling pathway, which governs diverse developmental processes and stress responses. Blueberry is an economically important berry-bearing crop and prefers to acidic soil. However, the understandings of *ARF* family has not yet been reported in blueberry.

**Results:**

In the present study, 60 *ARF* genes (*VcARF*) were identified in blueberry, and they showed diverse gene structures and motif compositions among the groups and similar within each group in the phylogenetic tree. Noticeably, 9 digenic, 5 trigenic and 6 tetragenic *VcARF* pairs exhibited more than 95% identity to each other. Computational analysis indicated that 23 *VcARF*s harbored the miRNA responsive element (MRE) of miR160 or miR167 like other plant *ARF* genes. Interestingly, the MRE of miR156d/h-3p was observed in the 5’UTR of 3 *VcARF*s, suggesting a potentially novel post-transcriptional control. Furthermore, the transcript accumulations of *VcARF*s were investigated during fruit development, and three categories of transcript profiles were observed, implying different functional roles. Meanwhile, the expressions of *VcARF*s to different pH conditions (pH4.5 and pH6.5) were surveyed in pH-sensitive and tolerant blueberry species, and a number of *VcARF*s showed different transcript accumulations. More importantly, distinct transcriptional response to pH stress (pH6.5) were observed for several *VcARF*s (such as *VcARF6*s and *VcARF19-3/19–4*) between pH-sensitive and tolerant species, suggesting their potential roles in adaption to pH stress.

**Conclusions:**

Sixty *VcARF* genes were identified and characterized, and their transcript profiles were surveyed during fruit development and in response to pH stress. These findings will contribute to future research for eliciting the functional roles of *VcARF*s and regulatory mechanisms, especially fruit development and adaption to pH stress.

**Supplementary Information:**

The online version contains supplementary material available at 10.1186/s12864-022-08556-y.

## Background

Blueberry (*Vaccinium* spp.) is an economically important small fruit crop widely cultivated all over the world. Blueberry fruit tastes sweet with variable acidity. More importantly, its fruit possesses human health-promoting effects due to the richness of active compounds (such as flavonoids, phenolic acids, and anthocyanins, which have been known as potent antioxidants), for example, vision improvement, anti-cancer activity, aging delay, and reduced risk of cardiovascular diseases [1]. Thus, considerable attention has been attracted to understand the regulation of its growth and development. To date, a number of players have been addressed to perform important functions in blueberry, including transcription factors (such as *VcSPL12*, *VcRR2*, *VcMYB*s, *VcFT*), miRNAs, and auxin-related gene *VcIAA27* [2–7]. Recently, high-throughput sequencing data provided important information for understanding the regulators that control blueberry growth and development [8–10]. However, our understandings of the regulatory network underlying its growth and development are extremely limited. More regulators need to be comprehensively identified and functionally addressed in blueberry.

The phytohormone auxin is involved in the regulation of almost all developmental processes in plants, and it governs gene expression through auxin signalling pathway. Auxin responsive factor (ARF) family is considered as one of core components in auxin signalling pathway [11]. Generally, ARF proteins comprise a DNA binding domain (DBD) at its N-terminal, an activation or repression domain at its middle region (MR), and dimerization domain (CTD) at its C-terminal [12]: the DBD is a critical domain for the recognition of the auxin-responsive element (AuxRE); the MR domain determines transcriptional activation or repression; the CTD domain is involved in the regulation of ARF activity by dimerizing with Aux/IAA family genes as well as between ARFs. Under low auxin levels, ARFs are generally under inactive status through formatting a complex with their inhibitor Aux/IAA proteins, therefore blocking the auxin signalling pathway. When auxin levels increase, however, Aux/IAA proteins can be directly bound by the SCF (TIR1/AFB) ubiquitin ligase, followed by protein degradation at the 26S proteasome. After release from Aux/IAA inhibition, ARFs can then activate or repress the expression of auxin-dependent genes. Thus, the Aux/IAA-ARF modules govern diverse processes of plant growth and development such as apical dominance, later root initiation and formation, and vascular differentiation, hypocotyl xylem expansion and cambium homeostasis, fruit development and maturation, cell division, expansion, and differentiation [11, 12]. For example, AtARF4 in Arabidopsis acts as a transcriptional repressor to regulate shoot regeneration through competing the interaction of AtIAA12 with AtARF5 [13]; MdARF13 in apple serves as a negative regulator of the anthocyanin metabolic pathway [14]; *AtARF7* and *AtARF19* confer the gravitropism and phototropism of plant hypocotyls through mediating the asymmetric expression of *AtSAUR* genes in Arabidopsis [15]; *MdARF8* facilitates lateral root formation in apple [16]. Noticeably, the Aux/IAA-ARF modules perform their functions in not only organ-dependent but also cell type-dependent way. For instance, the two complexes, AtJMJ30-AtARF and AtATXR2-AtARF, can alter the status of H3K9me3 and H3K36me3 at the loci of *AtLBD*s, respectively, therefore promoting their expressions during leaf-to-callus transition in Arabidopsis [17, 18]; tasiR-*ARF*s inhibits the expression of *AtARF3* in the hypodermal cells of Arabidopsis to repress the fate of ectopic megaspore mother cell [19]; *SlARF3* is involved in the formation of epidermal cells and trichome in tomato [20]; *AtARF5/MP* and its inhibitor *AtIAA12/BDL* enable to specify root cell in Arabidopsis [21].

As a critical component of the auxin signaling pathway, ARFs are encoded by a multigene family. The first *ARF* gene was isolated in Arabidopsis using a yeast one-hybrid screen, and several other *ARF*s were subsequently identified by sequence homology search or Y2H screen [22]. With the availability of genome and transcriptome sequences, *ARF* family have been identified and characterized in a number of plant species. For example, 31 *ARF* genes were identified in apple [23], 19 in grape and sweet orange [24, 25], 39 in poplar [26], 17 in physic nut [27], 89 in the three Apiaceae species (celery, coriander, and carrot) [28], 21 in tomato [29], and 25 in rice [30]. Emerging genetic and molecular evidences indicated that *ARF* family is likely responsible for the specificity of the auxin responses, and the distinct properties among *ARF* family might facilitate the generation of unique auxin response that triggers developmental process appropriately [31, 32]. The most direct evidence is that a number of *ARF* genes can serve as transcriptional activators, and some as repressors instead [33]. For instance, three *MpARF*s were identified in *Marchantia polymorpha*, and *MpARF1* and *MpARF2* were transcriptional activator and repressor, respectively, whereas *MpARF3* failed to show the activity of transcriptional activator and repressor [34]. Recently, DAP-Seq data provided a framework for understanding both specific and redundant aspects of ARF binding [35]. Thus, the identification and characterization of *ARF* family in distinct plant species is an essential step towards understanding their functional roles.

In blueberry, it has been reported that auxin might function in several processes such as the development of adventitious root, fruit growth, shoot proliferation, and leaf formation [4, 10, 36]. However, a comprehensive analysis of blueberry *ARF* (*VcARF*) family has not yet been reported. In the present study, 60 *VcARF* genes were genome-widely identified and characterized. Their gene structures, motif architectures, chromosomal locations, phylogenetic relationship were investigated. Furthermore, the miRNA responsive elements and *cis*-acting elements was computationally analyzed, and the transcript profiles of *VcARF*s were retrieved from publicly available data during fruit development and in response to pH stress. The findings will contribute to future research for addressing the functional roles of *VcARF* family, especially fruit development and adaption to pH stress.

## Results

### Characterization of *ARF* family genes in blueberry

To identify *ARF* family in blueberry, the CDS sequences of the *ARF* genes from apple, grape, tomato, Arabidopsis and rice were used as queries to conduct BLASTn against the database *Vaccinium corymbosum* GDV RefTrans V1. After the removal of redundant sequences, 60 *VcARF* genes were totally identified, which were correspondingly named in term of their homologs in Arabidopsis (Fig S1). It was reported that Arabidopsis *ARF*s were classified into five groups [37]. Noticeably, no *VcARF* was clustered into the class IV with inclusion of *AtARF12-15* and *AtARF20-23* (Fig S1).

The detailed information of all the 60 *VcARF*s is listed in Table S1. Briefly, the CDS lengths of *VcARF* family vary from 1698 to 6417, and their deduced proteins comprise 565–2138 amino acids with the estimated molecular weights from 62.77 to 236.27 kDa and the predicted protein isoelectric points from 5.46 to 8.61. All the VcARF proteins contain an Auxin_resp domain and a N-terminal B3-type DNA binding domain (DBD) that recognizes the auxin-responsive element (AuxRE), supporting that they are ARF proteins. The prediction of subcellular localization indicated that most of VcARF proteins are completely or mainly localized in the nuclear, whereas three large VcARFs are predominately distributed in the cytoplasm (VcARF2-2/2–3) or plasma membrane (VcARF2-1).

### Chromosomal localization of *VcARF* family genes and their evolutionary relationships

**Fig. 1:**
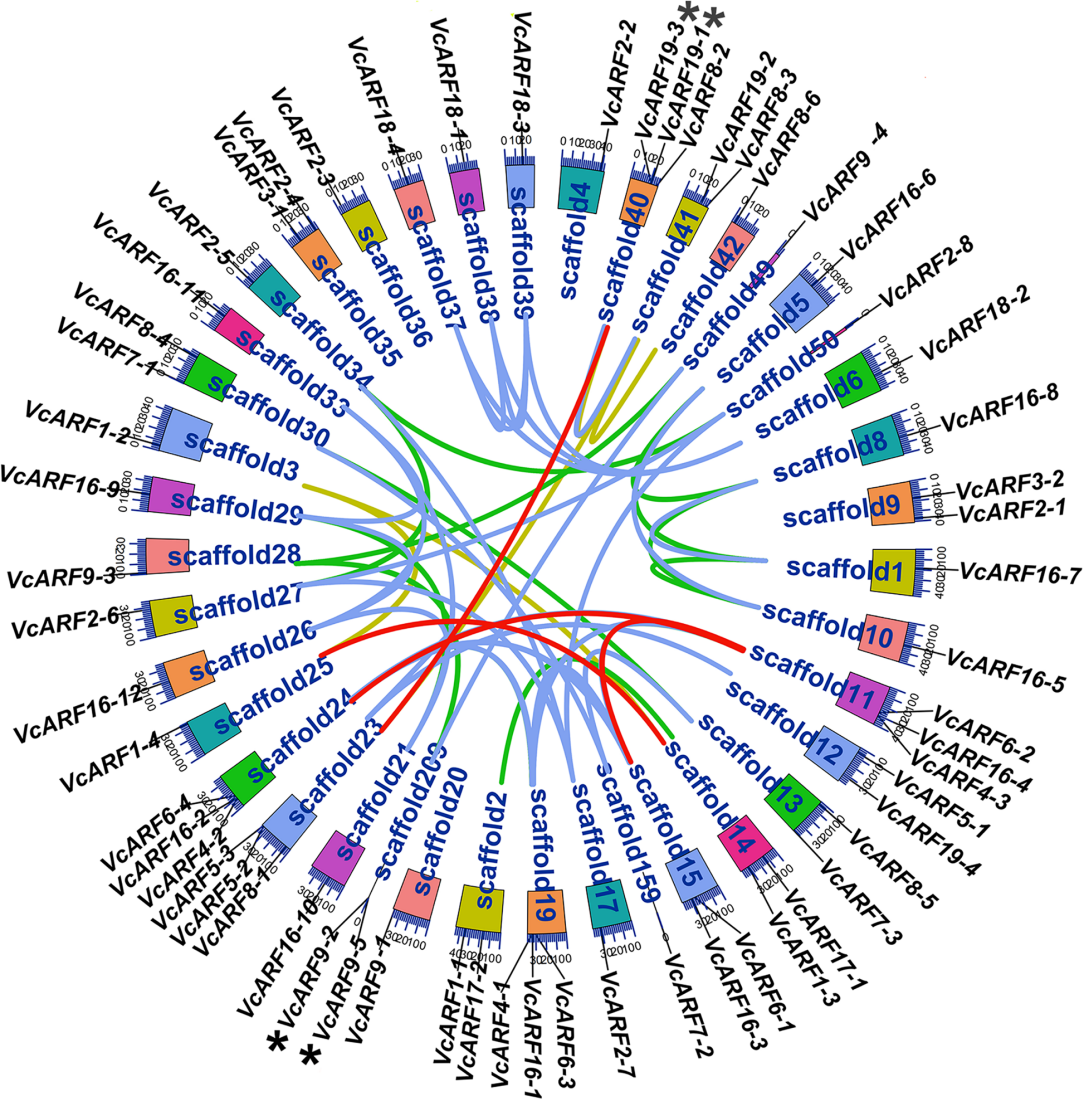
Chromosomal localization of the *VcARF* family genes. Each colored box represents a scaffold. The approximate distribution of each *VcARF* gene is marked on the circle with a short black line. The tandem duplicated genes are indicated with stars. Colored lines refer to the linkage group with high identity, red line 100%; blue line 99–100%; green line 98–99%; yellow-green 95–98%

To investigate the distribution of *VcARF* family genes in the draft genome (1760 scaffolds), a Circos map was built using the corresponding scaffolds where the *VcARF* genes are localized. Consequently, *VcARF*s are unevenly situated in 40 distinct scaffolds. As shown in Fig. 1, twenty five scaffolds harbor one *ARF* gene, while two *ARF* genes are separately distributed in each of 10 scaffolds and three *ARF*s in each of 5 scaffolds. Gene family can be generated from tandem duplication and segmental duplication of chromosomal regions as well as whole-genome duplication (WGD) [38, 39]. Tandem duplication is generally defined as two paralogs separated by the distance of 200 kb or less in the same chromosome [39]. It was observed that two pairs of *VcARF*s (*VcARF19-1* and *VcARF19-3*; *VcARF9-2* and *VcARF9-5*) are separated by 46.7 kb and 55.5 kb, respectively (Fig. 1). The identities at nucleotide level are separately 88% and 80% between *VcARF19-1* and *VcARF19-3* as well as *VcARF9-2* and *VcARF9-*5 (Table S2), suggesting that the gene pairs might be derived from tandem duplication. Meanwhile, it was noticed that 9 digenic, 5 trigenic and 6 tetragenic *VcARF* pairs show very high identity (more than 95%) to each other (Fig. 1 and Table S2), implying that they were possibly generated from segmental or whole-genome duplication. Furthermore, the non-synonymous/synonymous substitution ratio (Ka/Ks) was calculated between duplicated gene pairs. As shown in Table S2, the Ka/Ks values of all the gene pairs are less than 1 with the exception of two gene pairs (*VcARF19-1* and *VcARF19-3*, *VcARF18-2* and *VcARF18-5*), suggesting that these gene pairs and trigenic *VcARF*s have possibly undergone a purifying selection with restricted functional diversification.

**Fig. 2:**
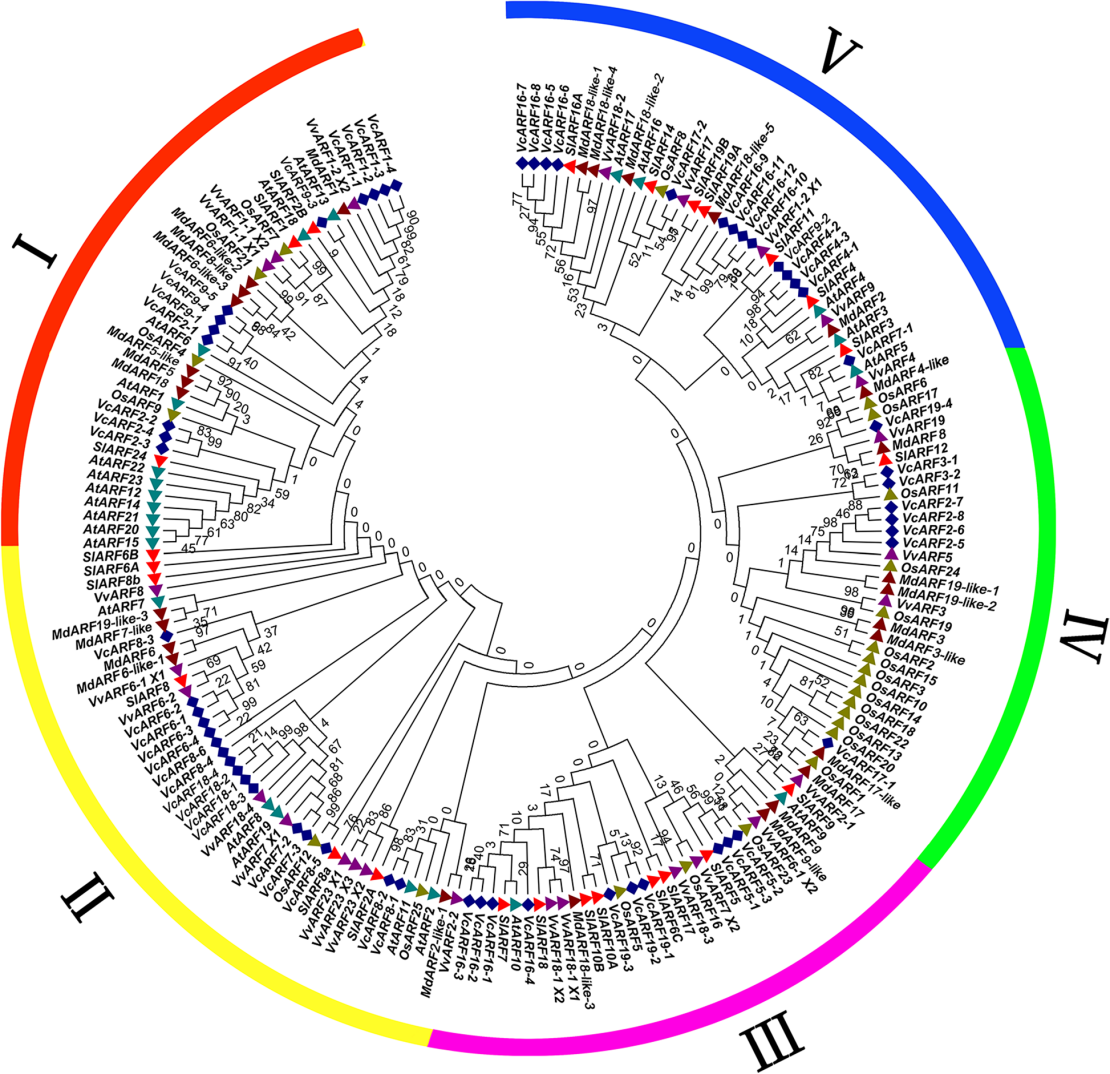
Phylogenetic relationship of *VcARF* genes with the *ARF*s in other plant species. The sequences of publicly known *ARF* genes in Arabidopsis (23), apple (28), grape (26), tomato (25) and rice (25) were downloaded from NCBI (www.ncbi.nlm.nih.gov), TAIR (www.arabidopsis.org) or RAP-DB (https://rapdb.dna.affrc.go.jp/) database. A maximum likelihood tree was generated with the CDS sequences of 187 *ARF* genes using the MEGA7 software, which were clustered into 5 groups (I-V). The numbers indicate the bootstrap values, which were calculated using 1000 replicates. The *ARF*s in the same species are represented with the same symbol: blue diamond, *Vaccinium corymbosum*; purple triangle, *Vitis vinifera*; dark purple triangle, *Malus domestica*; red triangle, *Solanum lycopersicum*; green triangle, *Arabidopsis thaliana*; light green triangle, *Oryza sativa*

To investigate their evolutionary relationship, phylogenetic analysis was performed using the CDS sequences of *VcARF*s and the *ARF*s from grape, apple, tomato, Arabidopsis and rice. Consequently, all the *ARF*s were classified into 5 different groups. *VcARF*s were separately distributed to the 5 groups and clustered together with *ARF*s from other plant species (Fig. 2). This observation suggested that *VcARF* family might have undergone evolutionary diversification similar to their counterparts from the other four plant species. Further observation indicated that a number of *VcARF*s were distributed at the same clade with function-known *ARF*s. For example, *VcARF5*s was grouped with *SlARF5* in the phylogenetic tree (Fig. 2), which affects fruit set and development through regulating auxin and gibberellin signaling in tomato [40]; loss of function of *SlARF4* clustered with *VcARF4*s confers the tolerance of water deficit in tomato [41].

### *VcARF* family shows diverse gene structures and motif compositions

**Fig. 3:**
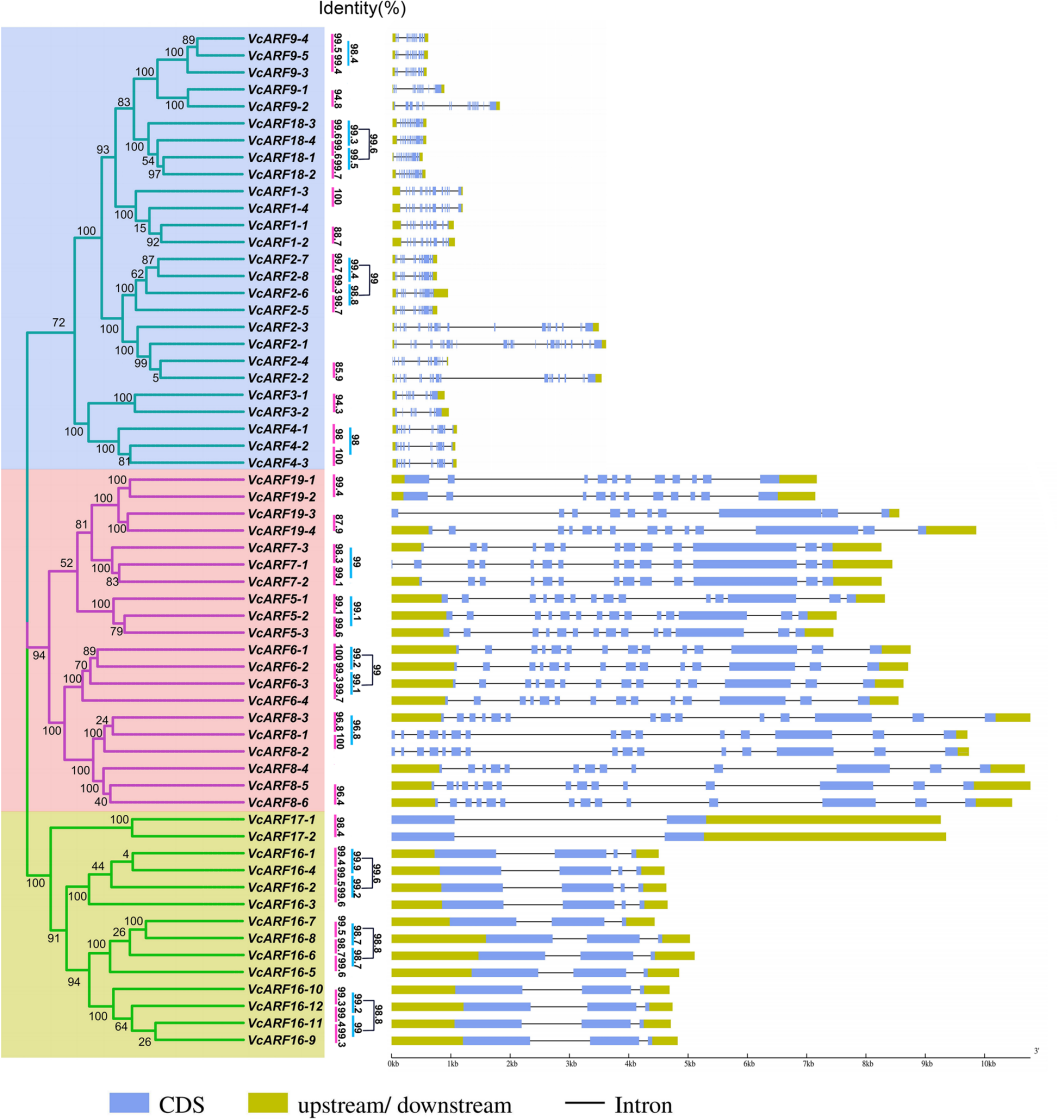
The exon/intron structures of *VcARF* genes. The left panel is the phylogenetic tree of *VcARF* genes. Three groups are clustered (I-III), and the identity between the gene pair is listed. The right panel shows the intron–exon structures where the exons are shown by blue rectangle, and the introns are represented by thin lines. Light green rectangle refers to upstream/downstream (5’UTR and 3’UTR)

Since the structural diversity provides important clues for gene duplication within gene family, the exon/intron structures were generated in terms of the gene coding and genomic sequences. It was observed that *VcARF* genes show a high variation in the number of exons. As shown in Fig. 3 and Table S1, forty two *VcARF*s have 10–16 exons with intron intervals. Fourteen *VcARF*s comprise only 2–4 exons including all the *VcARF16*s and *VcARF17*s, whereas a large number of exons exist in the genes *VcARF2-1*, *VcARF2-2*, *VcARF2-3* and *VcARF9-2* with the number of 32, 23, 25, 20, respectively (Fig. 3). Furthermore, integration analysis of phylogenetic relationship with gene structures was performed. Consequently, the *VcARF* pairs at the same clade in phylogenetic tree basically show similar exon/intron structures with high identity of more than 85% (Fig. 3). These observations supported that the *VcARF* pairs at the same clade might contribute to gene family expansion with less functional diversification.

**Fig. 4:**
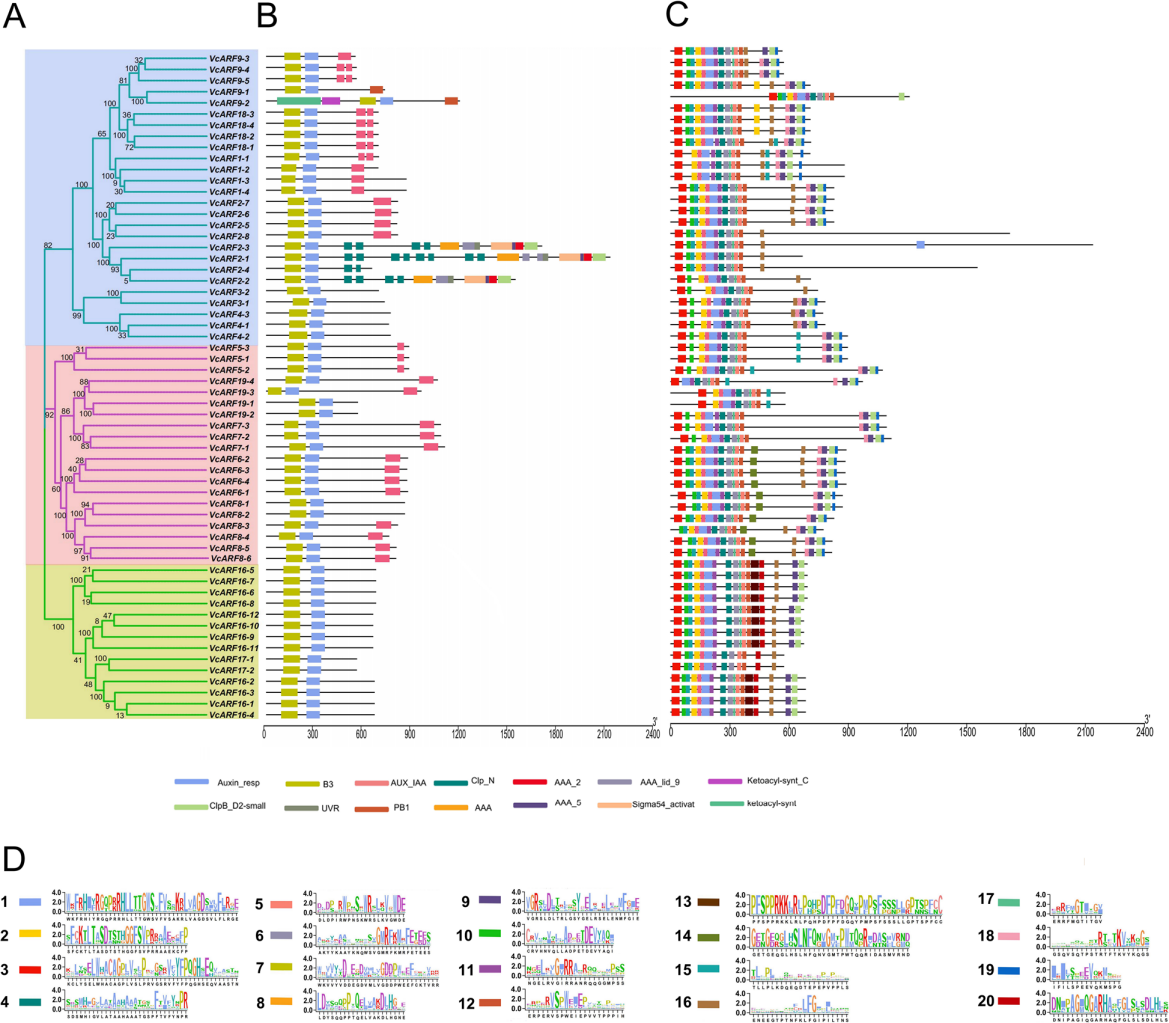
The conserved domains and motif compositions of VcARF proteins. (A) The phylogenetic tree of VcARF proteins, and three groups are clustered (I-III). (B) The conserved domains of VcARF proteins. Different colored rectangles represent different domains. (C) The motif compositions of VcARF proteins. Twenty motifs were identified using the program MEME, which are represented by the boxes of different colors. (D) The sequence logos of 20 motifs. The height of the letters within each stack indicates the relative frequency. The symbols are corresponding to the colored box in the right panel

To provide information regarding the functional diversity of VcARF family, the components of their motifs and domains were analyzed using the online tools MEME and Pfam. As a result, all the family members contain B3-type and Auxin_resp domains (Fig. 4). However, 27 VcARF proteins mainly at the class II and III lack the C-terminal AUX/IAA or PB1 domain functioning as a dimerization domain with AUX/IAA proteins, including VcARF3s, VcARF4s, VcARF16s, VcARF17s, VcARF2-1/2–2/2–3/2–4, VcARF8-1/8–2, VcARF19-1/19–2 (Fig. 4). This observation is consistent with previous reports that C-terminal AUX/IAA is missed in several ARF proteins in plants [28, 29, 37, 42]. Notably, additional domains were observed in five VcARF proteins (VcARF2-1/2–2/2–3/2–4/9–2). For example, VcARF2-1/2–2/2–3 contain Clp_N, ClpB_D2-small and three AAA-related domains which are related to binding, oligomerization or chaperone-like functions, while VcARF9-2 harbors Ketoacyl-synt_C and Ketoacyl-synt domains where the active site of beta-ketoacyl synthase is usually located (Fig. 4). Meanwhile, 20 motifs were identified for the 60 VcARFs. Although most of the VcARF proteins share the motifs 1, 2, 3, 4, 5, 6, 8, 10, diverse motif compositions exist in VcARF family (Fig. 4), implying their functional diversity. Further observation indicated that VcARF proteins at the same clade in the phylogenetic tree basically show similar motif composition (Fig. 4), suggesting a potential of functional redundancy.

### A group of *ARF* genes are targeted by miRNAs in blueberry

**Fig. 5:**
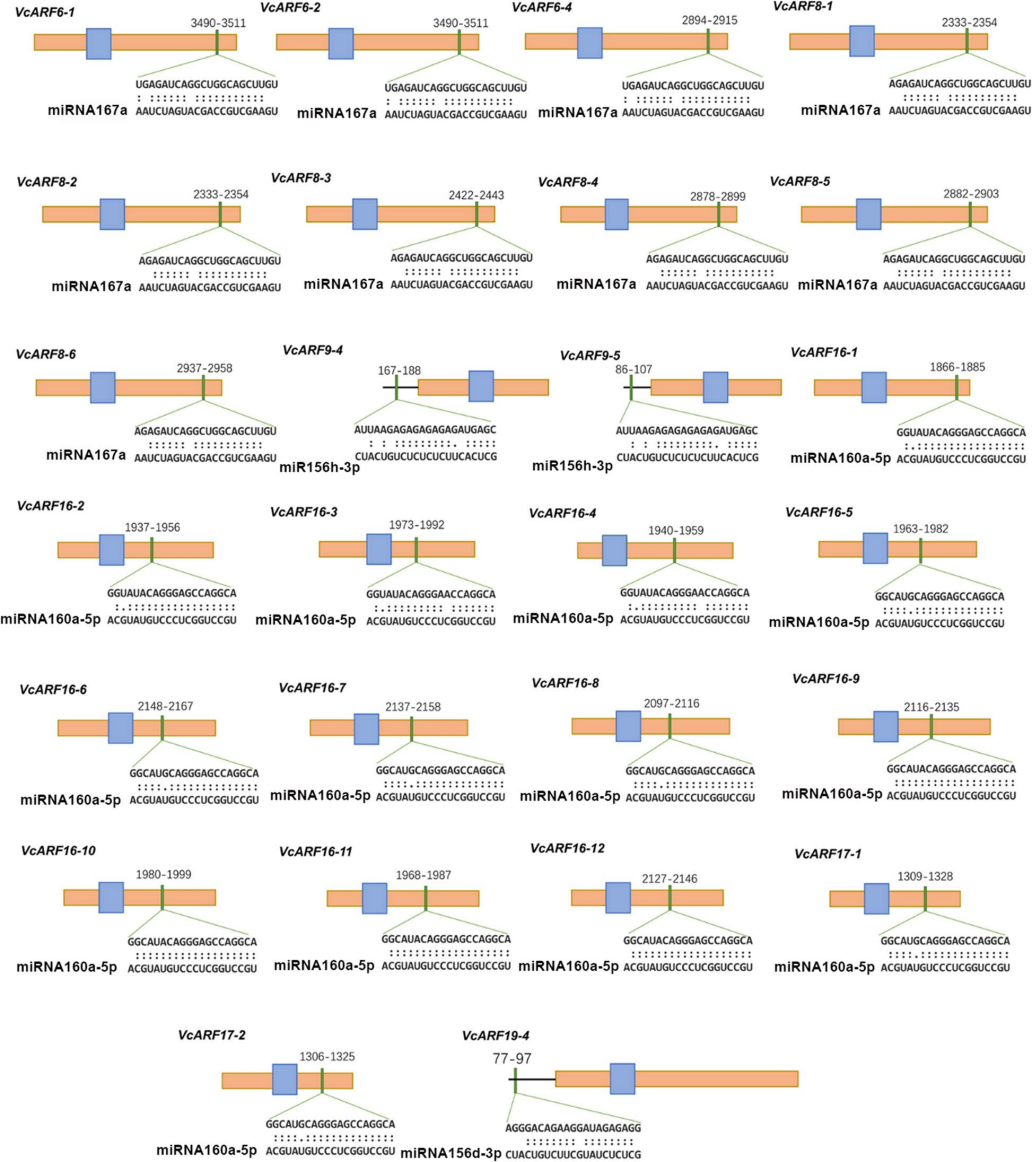
The diagrams of *VcARF* sequences targeted by miR167, miR160 or miR156. The orange box, black line, blue box, and green box represent CDS sequence, UTR region, ARF domain, and miRNA responsive element (MRE), respectively. The numbers indicate the localization of each MRE in the CDS sequence. Mature miRNA sequences are aligned along with their complementary sequences within *VcARF* genes, and the matched nucleotides are labeled with double dots

MicroRNAs plays important roles in the regulation of gene expression through mRNA cleavage or translational repression in plants. It has been well acknowledged that *ARF* genes can be targeted by miR160 and miR167 in plants [32]. To explore the *VcARF* family members potentially targeted by miRNAs, the miRNA responsive elements (MREs) were searched using the ORF sequences of 60 *VcARF*s against all the vco-miRNAs which were identified previously [3]. Consequently, 14 *VcARF*s (12 *VcARF16*s and 2 *VcARF17*s) harbor one MRE for miR160, while all the 6 *VcARF8*s and 3 *VcARF6*s (VcARF6-1/6–2/6–4) show one MRE for miR167 at the coding region (Fig. 5), which is in agreement with the previous reports that *ARF6/8/16/17* can be targeted by miR160 or miR167 [43, 44]. These observations suggested that they might have the potentials to be targeted by miR160 or miR167. Interestingly, the MRE for miR156d/h-3p is present in the 5’UTR of 3 *VcARF*s such as *VcARF9-4*, *VcARF9-5*, *VcARF19-4* (Fig. 5), suggesting that the post-transcriptional regulation of *ARF*s mediated by miR156-3p possibly exists in blueberry.

### *VcARF* family shows diverse transcript profiles during fruit development

**Fig. 6:**
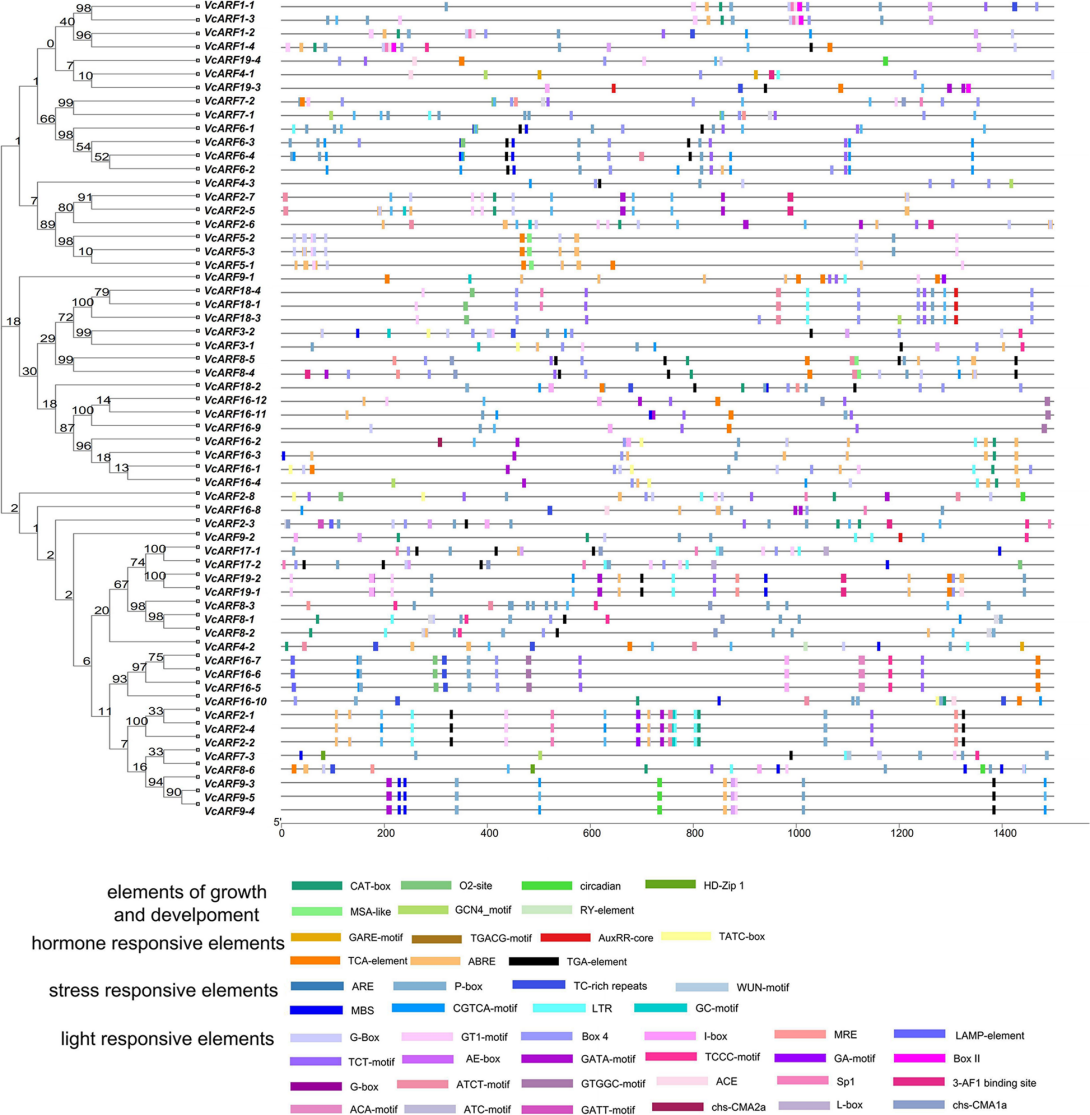
The *cis*-acting elements in the promoter regions of *VcARF* genes. The phylogenetic tree was generated using the promoter sequences of *VcARF* genes (the left panel), and the colored boxes in right panel represent different *cis*-acting elements. The symbols at the bottom are corresponding to the colored box in the right panel

To obtain hints for understanding the regulatory mode of gene expression, the *cis*-acting elements were predicted in the promoter regions of *VcARF*s (1500 bp upstream of the TSS) using the online program PlantCARE. As indicated in Fig. 6, besides the core elements, the *VcARF* family show 48 types of specific-function elements, including development-associated elements (for example RY-element, GCN4_motif, HD-ZIP), light responsive elements (such as G-box, TCT-motif, GT1-motif, AE-box, Box 4, GATA-motif, I-box, TCCC-motif, MRE, 3-AF1 binding site, chs-CMA1a, Box II, ATCT-motif, ACE, chs-CMA2a, Gap-box, Sp1), stress response elements (for example ARE, MBS, TC-rich repeats, LTR, GC-motif), phytohormone response element (such as ABRE, GARE-motif, P-box, TATC-box, TGACG-motif). Furthermore, phylogenetic analysis was performed using their promoter sequences. Interestingly, a number of promoters belonging to homologous *VcARF*s were clustered at the same clade in the phylogenetic tree, showing similar compositions of *cis*-acting elements (for example *VcARF16-5/16–6/16–7*, *VcARF9-3/9–4/9–5*) (Fig. 6). Generally, TGA-element and AuxRR-core are considered as auxin-related elements. It was observed that 25 *VcARF*s have the element TGA-element in their promoter regions, while the element AuxRR-core is present in the promoter regions of *VcARF9-1*, *VcARF9-3* and *VcARF9-4* (Fig. 6), supporting that they might be responsive to auxin activation or repression.

**Fig. 7:**
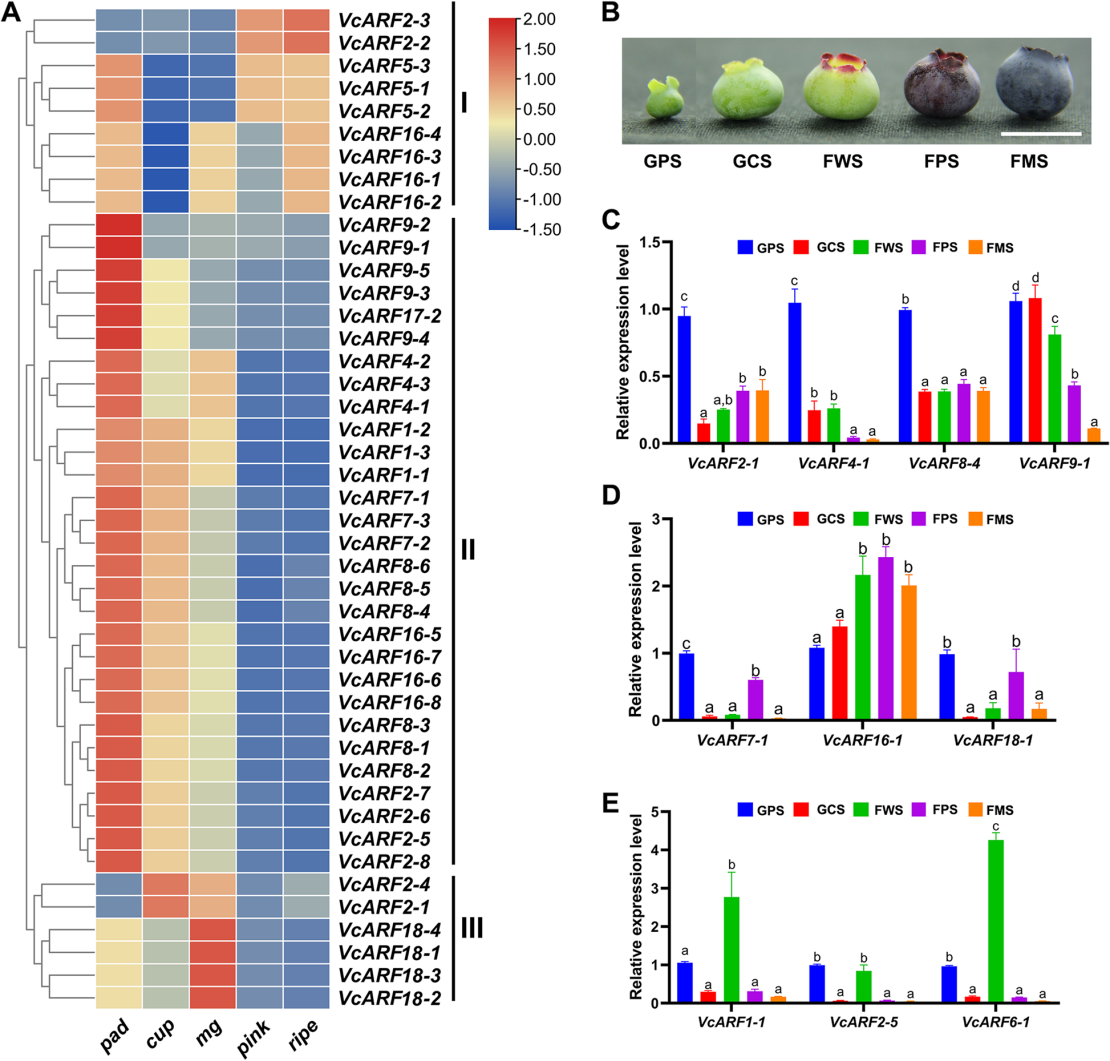
Expression analysis of *VcARF* genes during fruit development. **A** Transcript profiling of *VcARF*s during fruit development. The transcriptome data from different developmental stages (pad, cup, mg, pink, ripe) were extracted from the previous study reported by Gupta et al. [45]. The color scale beside the heat map indicates gene expression levels, low transcript abundance indicated by blue color and high transcript abundance indicated by red color. **B** Photograph of fruits at five developmental stages [green pad (GPS), green cup II (GCS), light green/white (FWS), pink (FPS) and blue (FMS) fruits], the scale bar indicates 1 cm. **C**-**E** Expression patterns of *VcARF*s during fruit development. Total RNAs were extracted from fruit of the cultivar ‘Northland’ (*V. corymbosum*) at the five above-mentioned developmental stages. Data were normalized against *VcACTIN*, and the expression level at GPS stage was set as 1. Error bars indicate SE of three biological and technical replicates, and different letters indicate significant difference (*P* < 0.05)

Blueberry fruit development can be generally divided into two phases: fruit growth and maturation. To understand the expression patterns of *VcARF*s during fruit development, we extracted the transcript profiling data of fruit at differently developmental stages including pad, cup, mature green, pink and ripe from the previous study reported by Gupta et al. [45]. The three early developmental stages represent the growth phase; while the pink and blue stages refer to the maturation phase. Consequently, the transcript profiling data of 44 *VcARF*s were obtained. As shown in Fig. 7A, they can be grouped into 3 classes based on their accumulation patterns. The class I comprises 9 *VcARF*s with relatively high transcript abundance at maturation stage. Notably, *VcARF2-2* and *VcARF2-3* were relatively low at the three early developmental stages, but then remarkably increased from pink to ripe stages (Fig. 7A). The class II consists of 29 *VcARF*s including *VcARF1*s, *VcARF4*s, *VcARF7*s, *VcARF8*s, *VcARF9*s, *VcARF17-2*, *VcARF2-5/2–6/2–7/2–8*, *VcARF16-5/16–6/16–7/16–8*, which were highly expressed at the pad stage and gradually decreased as fruit develops (Fig. 7A). The class III contains 6 *VcARF*s with relatively high accumulation at either cup or mature green stage such as *VcARF18*s and *VcARF2-1/2–4* (Fig. 7A). These transcript profiles implied that *VcARF* family might play different, even opposite, roles during fruit development.

Subsequently, ten *VcARF*s were chosen to investigate their expression patterns at five fruit developmental stages [green pad (GPS), green cup II (GCS), light green/white (FWS), pink (FPS) and blue (FMS) fruits] (Fig. 7B) using qRT-PCR approach, including *VcARF1-1*, *VcARF2-1*, *VcARF2-5*, *VcARF4-1*, *VcARF6-1*, *VcARF7-1*, *VcARF8-4*, *VcARF16-1*, *VcARF18-1*. As shown in Fig. 7C, the expressions of *VcARF2-1*, *VcARF4-1*, *VcARF8-4* and *VcARF9-1* were decreased as fruit develops, whereas *VcARF16-1* was transcriptionally increased when fruit ripens (Fig. 7D). Additionally, two genes *VcARF1-1*, *VcARF2-*5 showed relatively high expression at GPS and FPS stages (Fig. 7D), while maximum expression was observed at the FWS stage for the three genes *VcARF1-1*, *VcARF2-5*, *VcARF6-1* (Fig. 7E).

### *ARF* family exhibits different expression patterns in response to pH shift in blueberry

**Fig. 8:**
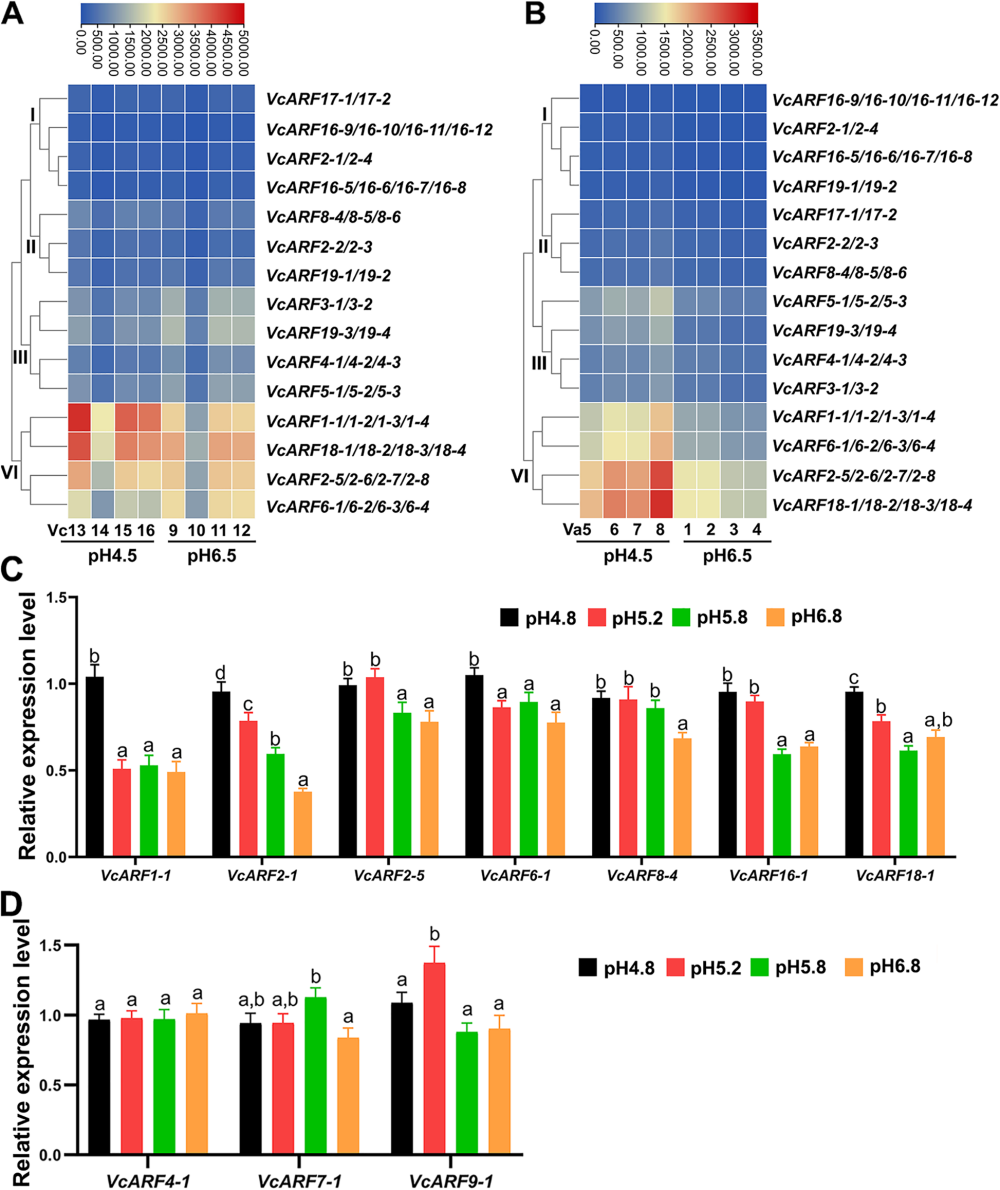
Expression analysis of *VcARF* genes in response to pH stress. **A**-**B** Transcript profiling of *VcARF*s in response to pH stress. The transcriptional response to different pH conditions (pH4.5 and pH6.5) in pH-sensitive *Vaccinium corymbosum* (**A**) and pH-tolerant *Vaccinium arboretum* (**B**). The transcriptome data were extracted from the publicly-available GDV database (http://www.vaccinium.org) for heatmap generation. Each of pH treatment has four samples for each species, and the numbers represent the sample IDs. The color scale above the heat map indicates gene expression levels, low transcript abundance indicated by blue color and high transcript abundance indicated by red color. **C**-**D** Expression patterns of *VcARF*s in response to pH stress. Total RNAs were extracted from tissue culture seedlings of the cultivar ‘Northland’ (*V. corymbosum*) under pH 4.8, 5.2, 5.8, 6.8. Data were normalized against *VcACTIN*, and the expression level under pH 4.8 was set as 1. Error bars indicate SE of three biological and technical replicates, and different letters indicate significant difference (*P* < 0.05)

Blueberry prefers to grow in acidic soils within the pH range from 4.0 to 5.3, and neutral to basic soil pH is usually harmful for its growth [36]. To explore the roles of *VcARF* family in response to pH stress, we retrieved the publicly available transcript profiling data of blueberry root (two species: pH-sensitive *Vaccinium corymbosum* and pH-tolerant *Vaccinium arboretum*) at its preferred pH of 4.5 and near neutral pH 6.5. According to their transcript accumulation patterns, the *VcARF*s in both species were classified into four groups (Fig. 8). The *VcARF*s in the groups I and II showed relatively low and stable expression in different pH conditions. However, altered transcript accumulation was observed for the *VcARF*s in the group III and IV (Fig. 8). In pH-sensitive *V. corymbosum*, *VcARF1*s and *VcARF18*s showed relatively high transcript accumulation under the condition of preferred pH (4.5), whereas relatively high accumulation was observed for *VcARF3*s, *VcARF19-3* and *VcARF19-4* at near neutral pH 6.5 (Fig. 8A). In pH-tolerant *V. arboretum*, the *VcARF*s in the group III and IV (such as *VcARF1*s, *VcARF6*s, *VcARF6*s, *VcARF18*s, *VcARF2-5/2–6/2–7/2–8*, *VcARF19-3/19–4*) were suppressed by near neutral pH 6.5 (Fig. 8B). When compared the accumulation patterns of *VcARF* genes between pH-sensitive and pH-tolerant species, it was found that *VcARF1*s and *VcARF18*s displayed similar accumulation patterns in both species (Fig. 8). Interestingly, several *VcARF*s showed different, even opposite, accumulation patterns. For example, *VcARF6*s and *VcARF19-3/19–4* in pH-tolerant *V. arboretum* were depressed when the pH shifted from 4.5 to 6.5 (Fig. 8B), whereas the converse patterns were observed for them in pH-sensitive *V. corymbosum* (Fig. 8A).

Increasing evidence indicated that above-ground plant parts can quickly respond to pH stress via regulating its growth and signal exchange between leaf and root [46]. To explore how *ARF* genes affect the adaption of above-ground tissues to pH switch, the young leaves of tissue culture seedlings were used to investigate their responses to pH shift (pH 4.8, 5.2, 5.8, 6.8) in *V. corymbosum*. Ten *VcARF*s were chosen to perform qRT-PCR analysis, including *VcARF6-1*, *VcARF7-1*, *VcARF16-1*, *VcARF9-1* and six genes with publicly available transcript profiling (*VcARF1-1*, *VcARF2-1*, *VcARF2-5*, *VcARF4-1*, *VcARF8-4*, *VcARF18-1*). As shown in Fig. 8C, seven genes *VcARF1-1*, *VcARF2-1*, *VcARF2-5*, *VcARF6-1*, *VcARF8-4*, *VcARF16-1*, *VcARF18-1* were transcriptionally decreased as pH shifted from 4.8 to 6.8. Especially, the expression levels of *VcARF1-1* and *VcARF2-1* at pH 5.2, 5.8, 6.8 were 47.2%-50.8% and 39.5%-82.3% of the ones at pH 4.8, respectively. In contrast, an enhanced expression was observed only at pH 5.2 and 5.8 for *VcARF7-1* and *VcARF9-1*, respectively (Fig. 8D). No significant change was observed for *VcARF4-1*. When compared with their publicly available transcript profiling, 5 out of 6 genes (*VcARF1-1*, *VcARF2-5*, *VcARF4-1*, *VcARF8-4*, *VcARF18-1*) showed similar tendency between expression pattern and transcript profiling in *V. corymbosum* (Fig. 8A and 8C-8D). These observations suggested that *VcARF* family, at least a subset of *VcARF* genes, might be involved in the response to pH stress.

## Discussion

Plant *ARF* genes act as key components of auxin signaling pathway to mediate the expression of auxin-responsive genes [11, 12]. In the present study, 60 *VcARF* genes were identified in blueberry, and the number of *VcARF* family members is bigger than the ones in apple (28), grape (26), Arabidopsis (23), sweet orange (19), and poplar (21) [23–26, 37]. The genome size (haploid) of blueberry is approximately 600 Mb [47], and the annotation of this genome has provided a nonredundant set of 56,087 genes [45], which is roughly 2.05 times as many genes as were annotated in Arabidopsis (27,411, TAIR10). Thus, it is reasonable for blueberry to have a large number of *ARF* genes. Phylogenetic analysis indicated that *VcARF*s can be clustered together with the *ARF*s from other plant species (Fig. 2), suggesting they might have undergone similar evolutionary diversification. Noticeably, 9 digenic, 5 trigenic and 6 tetragenic *VcARF* pairs showed very high identity (more than 95% at nucleotide level) to each other (Fig. 1 and Table S2), implying that they might have restricted functional diversification. Gene family generally arises from gene duplication during evolution, therefore leading to the acquisition of neofunctionalizations and the emergence of backup or redundant genes [48]. It was estimated that at least three rounds of whole-genome duplications occurred during the evolution of blueberry species [9], which is supposed to facilitate the generation of multiple copy genes. Thus, we assumed that the duplication events of chromosomal segment and whole genome are primarily responsible for the expansion of *ARF* genes in blueberry.

Considerable evidences indicated that *ARF*s play important roles during fruit growth as key components of auxin signaling pathway. For example, *SlARF10* and *SlARF6A* facilitate chlorophyll accumulation through activating the expression of *SlGLK1* [49, 50], while *MdARF106* in 'Royal Gala' apples is expressed during cell division and expansion, suggesting a potential role in the regulation of fruit size [51]. Previously, we revealed that *VcARF18* exhibited high transcript accumulation at fruit early developmental stages and remarkably declined at the initiation of fruit maturation [3]. In the present study, 29 *VcARF*s showed relatively high expression at the pad stage, and gradually decreased as fruit grows and ripens (Fig. 7). These observations suggested that *VcARF*s possibly exert functions during fruit growth. Fruit maturation can be accompanied by a series of cellular, molecular, biochemical, and structural changes [42]. It has been documented that *ARF* genes are involved in the regulation of fruit maturation. For example, *MdARF5* activates the expression of ethylene-related genes (*MdERF2*, *MdACS*s, *MdACO*s) to initiate fruit maturation in apple [52]; *CpARF2* is implicated in the mediation of papaya fruit maturation by promoting the transcriptional activity of *CpEIL1* (a key component of ethylene signaling) and the stabilization of CpEIL1 protein [53]; *MdARF13* can negatively regulate anthocyanin accumulation of apple fruit directly through repressing the expression of *MdDFR* [54]. In the present study, 9 *VcARF*s showed high transcript abundance at maturation stage as compared to early developmental stages, implying a potential role during fruit maturation. Intriguingly, the previous data provided by Gupta et al. [45] indicated that the expressions of three genes (*VcACO*, CUFF.8159.1, *VcEIL1*, CUFF.53576; *VcDFR*, CUFF.38755) were up-regulated as fruit develops and ripens, whereas the chlorophyll-related gene *VcGLK1* was transcriptionally down-regulated (Fig S2). These results implied that co-expression pattern might exist between *VcARF*s and their targets or downstream genes. Thus, the transcript profiles of *VcARF*s supported that *ARF* genes may exert important functions in the regulation of fruit growth and maturation.

Blueberry prefers to grow in acidic soils, and neutral to basic pH is generally stressful to the commonly cultivated blueberry species [36]. *ARF*s are considered as key regulators to respond to various stresses [11, 12]. However, it is unclear if *ARF*s perform functions in plant response to pH stress. In the present study, similar accumulation patterns were observed for several *ARF*s (*VcARF1*s and *VcARF18*s) in pH-sensitive and pH-tolerant blueberry species (Fig. 8), suggesting some common roles of *ARF* family in response to pH stress in different species. Noticeably, a group of *VcARF*s showed different, even opposite, transcriptional response to pH stress between pH-sensitive and pH-tolerant species (Fig. 8), implying that they might contribute to different tolerance capacity to pH stress. Previous studies indicated that plant adaptation to high pH soil requires an effective transcriptional regulation mainly associated with nutrition balance, ROS-mediated responses, detoxification and cell wall [36, 55]. It has been addressed that *AtARF2* acts as transcriptional repressor to participate in the regulation of K^+^ uptake through mediating *AtHAK5* transcription in Arabidopsis [56]; *OsARF16* is required for iron deficiency response in rice [57]; *AtARF3/ETT* is a positive player in regulating the activity of pectin methylesterase (PME) in the cell wall of Arabidopsis [58]; loss of function of *SlARF4* confers plant tolerance to water deficit by enhancing Superoxide Dismutase (SOD) and antioxidant substances in tomato [41]. It is possible that the transcriptional alteration of *VcARF*s under different pH conditions might be required for adjusting nutrient availability, ROS-mediated responses, detoxification or/and cell wall. Also, it is worth to explore if *VcARF*s are involved in the adjustment of the internal pH value. Interestingly, the previous transcriptomic study indicated that all the three genes (*VcHAK*, CUFF.225.1; *VcPME*, CUFF.59080.1; *VcSOD*, CUFF.32906.1) were transcriptionally suppressed in pH-tolerant species under the condition of pH 6.5 (Fig. S2) [59]. Thus, we proposed an assumption that *ARF* family in blueberry might be involved in response to pH stress possibly via affecting nutrition balance, ROS-mediated responses, detoxification and cell wall.

## Conclusions

In conclusion, 60 *ARF* genes were identified and characterized in blueberry. The combination analysis of gene structures, motif architectures, phylogenetic relationship, sequence identity and miRNA responsive elements suggested their conservation and divergence in features and functional roles across plant species. Furthermore, we assumed that *VcARF*s might have potential roles during fruit development and in response to pH stress based on their transcript profiles. These findings will contribute to future research for documenting the functional roles of *ARF*s and their regulatory mechanisms in blueberry, especially fruit development and adaption to pH stress.

## Materials and methods

### Identification of *VcARF* family genes

The sequences of publicly known *ARF* genes in Arabidopsis, apple, grape, tomato and rice were downloaded from NCBI (www.ncbi.nlm.nih.gov), TAIR (www.arabidopsis.org) or RAP-DB (https://rapdb.dna.affrc.go.jp/) database. All the downloaded sequences were applied to BLASTn search against *Vaccinium corymbosum* cv. Draper genome v1.0 (www.vaccinium.org), and all the potential hits to the conserved regions of *ARF* genes were subsequently collected. Furthermore, redundant sequences were removed from all the potential hits, and then the complete transcript sequences were retrieved from the database of *Vaccinium corymbosum* GDV RefTrans v1 (www.vaccinium.org). Each of the predicted VcARF proteins was applied to a confirmation of auxin response factor using the online program Pfam (http://pfam.xfam.org/). The subcellular localizations of VcARF proteins were predicted using the online program WoLF pSORT (https://wolfpsort.hgc.jp/).

### Chromosomal locations of *VcARF*s and phylogenetic analysis of *ARF* genes

All the 60 *VcARF* genes were mapped to the genome of *Vaccinium corymbosum* (Draper), and their location information was obtained accordingly. Subsequently, the information regarding their positions, the correspondingly situated scaffolds, and identity (95%) were imported into the software TBtools to generate a circle plot [60]. The CDS sequences of *ARF* genes (26 from grape, 28 from apple, 23 from Arabidopsis, 25 from tomato, and 25 from rice) were applied to generate phylogenetic tree with the above 60 *VcARF*s. Briefly, multiple sequences alignment of all the *ARF* genes were performed by Clustal X, and phylogenetic tree was constructed by the MEGA7.0 software with maximum likelihood statistical method [61]. Bootstrap values were calculated using 1000 replicates.

### Gene structures, conserved motifs and domains of VcARFs

The exon/intron structures of 60 *VcARF* genes were drawn using GSDS 2.0 (http://gsds.gao-lab.org/) through comparing their genomic sequences and coding sequences. The conserved motifs of VcARF proteins were analyzed using the online program Multiple Expectation Maximization for Motif Elucidation (http://meme-suite.org/tools/meme). The conserved domains were analyzed using the online program Pfam (http://pfam.xfam.org/).

### Promoter analysis of *VcARF* genes

The transcription start sites (TSS) of *VcARF*s were predicted by the online program TSSP in Softberry (http://linux1.softberry.com/berry.phtml?topic=tssp&group=-programs&subgroup=promoter). Subsequently, a 1,500 bp interval upstream of the TSS was considered as promoter and applied to the online program PlantCARE (http://bioinformatics.psb.ugent.be/webtools/plantcare/html/) for promoter analysis.

### Prediction of miRNA-targeted *VcARF*s

The online programe psRNATarget (http://plantgrn.noble.org/psRNATarget/home) was used to predict miRNA-targeted *VcARF*s. Briefly, the sequences of sixty *VcARF*s and blueberry miRNAs identified previously [3] were submitted to psRNATarget, and the parameters were set as default with a maximum expectation value of 3.0. The diagrams were drawn based on the gene structure accordingly.

### Expression profiling of *VcARF*s during fruit development and in response to pH stress

To understand the expression patterns of *VcARF* genes in response to pH stress, their publicly available transcript profiling data [59] were retrieved at the database GDV (www.vaccinium.org) regarding blueberry root (two species: pH-sensitive *Vaccinium corymbosum* and pH-tolerant *Vaccinium arboretum*) at its preferred pH of 4.5 and near neutral pH 6.5. Briefly, the transcript ID of each *ARF* gene was obtained using sequence search. Subsequently, the transcript profiling data were retrieved using the online program “Expression Heatmap” (www.vaccinium.org/node/854703). The transcript profiling data of *VcARF*s during fruit development (five developmental stages such as pads, cups, green, pink, and ripe) were extracted from the previous study reported by Gupta et al. [45]. All the heatmaps were generated using the software TBtools [60].

The pH of the woody plant medium (WPM, PhytoTech, USA) was adjusted using 1 M KOH until a desired final pH 4.8, 5.2, 5.8, or 6.8. Five young tissue culture seedlings with similar growth were transferred to the WPM medium with different pHs, and five Petri plates were used for each treatment. The seedlings with different pH treatment were grown for two weeks under the condition of 16/8 h photoperiod at 20 °C. Subsequently, young leaves of the treated seedlings were collected for RNA extraction.

Total RNAs were extracted from fruit at five developmental stages [green pad (GPS), green cup II (GCS), light green/white (FWS), pink (FPS) and blue (FMS) fruits] and young leaves of tissue culture seedlings of *V. corymbosum* (cultivar ‘Northland’) under pH 4.8, 5.2, 5.8, 6.8. cDNAs were synthesized using StarScript II First-strand cDNA Synthesis Mix With gDNA Remover Kit (GenStar, China). qRT-PCR was conducted using the Bio-Rad CFX Connect Real-Time PCR Detection System with the reagent of 2 × RealStar Green Fast Mixture (GenStar, China). Data analysis were performed using the software Bio-Rad CFX Manager, and *VcACTIN* was set as an internal reference for data normalization. Three biological replicates for each sample were conducted with three technical replicates, and error bars indicate SE. Statistical significance of the data was analyzed using ANOVA with LSD test, and *p*-value < 0.05 was considered to be statistically significant. Primer information is listed in Table S3.

## Supplementary Information

**Additional file 1:** **Table S1.** Characterization of the *ARF* gene familyin blueberry.

**Additional file 2:** **Table S2.** Identities of VcARF gene pairs and their corresponding Ka/Ks values.

**Additional file 3:** **Table S3.** Primers used in the study

**Additional file 4:** **Figure S1.** Phylogenetic analysis of ARF genes in blueberry and Arabidopsis. The CDS sequences of AtARFs were downloaded from the TAIR website (www.arabidopsis.org). A maximum likelihood tree was generated with the CDS sequences of the ARF genes using the MEGA7 software. 

**Additional file 5:** **Figure S2.**  Transcript profiling of the potential targets or downstream genes of VcARFs. (A) Transcript profiling during fruit development (five stages: pad, cup, mg, pink, ripe). (B-C) Transcript profiling in response to different pH conditions (pH4.5 and pH6.5) in pH-sensitive Vaccinium corymbosum  (B) and pH-tolerant Vaccinium arboretum (C). The color scale beside the heat map indicates gene expression levels, low transcript abundance indicated by blue color and high transcript abundance indicated by red color. The heatmaps were generated using the software TBtools (Version 1.098689, https://github.com/CJ-Chen/TBtools/releases). 

**Additional file 6.** Raw data for qRT-PCR investigation during fruit development.

## Data Availability

The publicly available transcript profiling data regarding the response to different pH were retrieved at the database GDV (www.vaccinium.org). All data generated or analysed during this study are included in this published article [and its supplementary information files].
