# Supplementary material for: Identification of ARF family in blueberry and its potential involvement of fruit development and pH stress response
Source: BMC Genomics. 2022 Apr 27;23:329. doi: 10.1186/s12864-022-08556-y (PMC9047364; doi:10.1186/s12864-022-08556-y)
Supplement: Supplementary file 1 — Additional file 1: Table S1. Characterization of the ARF gene familyin blueberry. [file 12864_2022_8556_MOESM1_ESM.docx]

**Table S1** Characterization of the *ARF* gene family in blueberry.

| **ID** | **Name** | **Scaffold** | **Location** | **CDS length (bp)** | **Protein** | | | **Exon** | **Subcellular localization** |
| --- | --- | --- | --- | --- | --- | --- | --- | --- | --- |
|  |  |  |  |  | Size (aa) | MW (kDa) | pI |  |  |
| 1 | *VcARF1-1* | 2 | VaccDscaff2:35513223..35522595+ | 2133 | 710 | 78.86 | 5.78 | 14 | nucl/chlo/plas/cyto |
| 2 | *VcARF1-2* | 3 | VaccDscaff3:5089913..5099455- | 2121 | 706 | 78.33 | 6.11 | 14 | nucl/chlo/cyto/mito |
| 3 | *VcARF1-3* | 14 | VaccDscaff14:30285241..30295980+ | 2643 | 880 | 98.11 | 6.94 | 16 | nucl/chlo |
| 4 | *VcARF1-4* | 25 | VaccDscaff25:35261834..35272570+ | 2643 | 880 | 98.11 | 6.94 | 16 | nucl/chlo |
| 5 | *VcARF2-1* | 9 | VaccDscaff9:37033642..37066587- | 6417 | 2138 | 236.27 | 8.33 | 32 | plas/cyto/nucl |
| 6 | *VcARF2-2* | 4 | VaccDscaff4:39140723..39172700- | 4662 | 1553 | 172.04 | 7.79 | 23 | cyto/nucl/chlo/plas |
| 7 | *VcARF2-3* | 36 | VaccDscaff36:3963855..3995414+ | 5154 | 1717 | 190.21 | 8.23 | 25 | cyto/nucl/chlo/plas |
| 8 | *VcARF2-4* | 35 | VaccDscaff35:28655149..28663653- | 2004 | 667 | 73.97 | 8.61 | 16 | nucl |
| 9 | *VcARF2-5* | 34 | VaccDscaff34:6479810..6486650+ | 2469 | 822 | 91.86 | 6.14 | 14 | nucl |
| 10 | *VcARF2-6* | 17 | VaccDscaff27:27973739..27982227- | 2487 | 828 | 92.41 | 6.21 | 14 | nucl |
| 11 | *VcARF2-7* | 17 | VaccDscaff17:31429406..31436228- | 2484 | 827 | 92.36 | 6.21 | 14 | nucl |
| 12 | *VcARF2-8* | 50 | VaccDscaff50:1263534..1270333- | 2484 | 827 | 92.35 | 6.21 | 14 | nucl |
| 13 | *VcARF3-1* | 35 | VaccDscaff35:11275837..11283809+ | 2238 | 745 | 82.05 | 6.89 | 11 | nucl |
| 14 | *VcARF3-2* | 9 | VaccDscaff9:16724921..16733537+ | 2130 | 709 | 78.06 | 7.05 | 10 | nucl |
| 15 | *VcARF4-1* | 19 | VaccDscaff19:33590293..33600143+ | 2316 | 771 | 85.37 | 6.09 | 12 | nucl |
| 16 | *VcARF4-2* | 24 | VaccDscaff24:9683280..9692882- | 2349 | 782 | 86.68 | 6.21 | 12 | nucl |
| 17 | *VcARF4-3* | 11 | VaccDscaff11:30535118..30544916+ | 2349 | 782 | 86.67 | 6.21 | 12 | nucl |
| 18 | *VcARF5-1* | 12 | VaccDscaff12:5638023..5646179+ | 2691 | 896 | 99.75 | 5.46 | 14 | nucl |
| 19 | *VcARF5-2* | 23 | VaccDscaff23:34950200..34957557+ | 2691 | 896 | 99.64 | 5.46 | 14 | nucl |
| 20 | *VcARF5-3* | 23 | VaccDscaff23:35564675..35571979- | 2691 | 896 | 99.80 | 5.46 | 14 | nucl |
| 21 | *VcARF6-1* | 15 | VaccDscaff15:25800314..25808896+ | 2670 | 889 | 98.20 | 6.26 | 14 | nucl/cyto |
| 22 | *VcARF6-2* | 11 | VaccDscaff11:27366853..27375393+ | 2670 | 889 | 98.17 | 6.20 | 14 | nucl/cyto |
| 23 | *VcARF6-3* | 19 | VaccDscaff19:30557305..30565768+ | 2655 | 884 | 97.56 | 6.09 | 14 | nucl/cyto |
| 24 | *VcARF6-4* | 24 | VaccDscaff24:12687653..12696034- | 2655 | 884 | 97.60 | 6.12 | 14 | nucl/cyto |
| 25 | *VcARF7-1* | 30 | VaccDscaff30:295030..303309+ | 3354 | 1117 | 124.71 | 6.64 | 14 | nucl/chlo/plas/cyto |
| 26 | *VcARF7-2* | 159 | VaccDscaff159:274810..282910+ | 3282 | 1093 | 121.88 | 6.39 | 13 | nucl/cyto |
| 27 | *VcARF7-3* | 13 | VaccDscaff13:39198442..39206540- | 3279 | 1029 | 121.78 | 6.26 | 13 | nucl/cyto |
| 28 | *VcARF8-1* | 23 | VaccDscaff23:4968620..4978140+ | 2613 | 870 | 96.86 | 5.81 | 15 | nucl/mito |
| 29 | *VcARF8-2* | 40 | VaccDscaff40:26970680..26980225+ | 2613 | 870 | 96.86 | 5.81 | 15 | nucl/mito |
| 30 | *VcARF8-3* | 41 | VaccDscaff41:25169999..25180560+ | 2484 | 827 | 92.05 | 5.75 | 14 | nucl |
| 31 | *VcARF8-4* | 30 | VaccDscaff30:28538840..28549308+ | 2322 | 773 | 86.06 | 5.83 | 13 | nucl/chlo/vacu |
| 32 | *VcARF8-5* | 13 | VaccDscaff13:2964750..2975312+ | 2457 | 818 | 91.21 | 5.88 | 15 | nucl |
| 33 | *VcARF8-6* | 42 | VaccDscaff42:3134356..3144615- | 2451 | 816 | 90.89 | 5.82 | 14 | nucl |
| 34 | *VcARF9-1* | 20 | VaccDscaff20:36291973..36299899+ | 2244 | 747 | 84.17 | 7.97 | 14 | nucl/cyto |
| 35 | *VcARF9-2* | 209 | VaccDscaff209:118212..134625- | 3630 | 1209 | 132.92 | 8.22 | 20 | nucl/chlo |
| 36 | *VcARF9-3* | 28 | VaccDscaff28:1273882..1279100- | 1698 | 565 | 63.20 | 5.55 | 14 | nucl/chlo |
| 37 | *VcARF9-4* | 49 | VaccDscaff49:1835768..1841224- | 1719 | 572 | 64.00 | 5.62 | 14 | nucl/chlo |
| 38 | *VcARF9-5* | 209 | VaccDscaff209:57292..62704- | 1719 | 572 | 64.00 | 5.62 | 14 | nucl/chlo |
| 39 | *VcARF16-1* | 19 | VaccDscaff19:33308214..33312630+ | 2052 | 683 | 75.38 | 7.85 | 4 | nucl |
| 40 | *VcARF16-2* | 24 | VaccDscaff24:9951498..9956041- | 2052 | 683 | 75.38 | 7.85 | 4 | nucl |
| 41 | *VcARF16-3* | 15 | VaccDscaff15:28595844..28600407+ | 2052 | 683 | 75.51 | 7.52 | 4 | nucl |
| 42 | *VcARF16-4* | 11 | VaccDscaff11:30200121..30204632+ | 2052 | 683 | 75.42 | 7.52 | 4 | nucl |
| 43 | *VcARF16-5* | 10 | VaccDscaff10:22380725..22385479- | 2079 | 692 | 76.29 | 6.41 | 3 | nucl |
| 44 | *VcARF16-6* | 5 | VaccDscaff5:17374973..17379982- | 2079 | 692 | 76.31 | 6.32 | 3 | nucl/cyto |
| 45 | *VcARF16-7* | 1 | VaccDscaff1:18439481..18443830- | 2079 | 692 | 76.35 | 6.38 | 3 | nucl/cyto |
| 46 | *VcARF16-8* | 8 | VaccDscaff8:23759116..23764049+ | 2079 | 692 | 76.32 | 6.30 | 3 | nucl |
| 47 | *VcARF16-9* | 29 | VaccDscaff29:17166955..17171684- | 2025 | 674 | 74.34 | 6.06 | 3 | nucl |
| 48 | *VcARF16-10* | 21 | VaccDscaff21:17724006..17728602- | 2025 | 674 | 74.48 | 5.98 | 3 | nucl |
| 49 | *VcARF16-11* | 33 | VaccDscaff33:17097454..17102070+ | 2025 | 674 | 74.27 | 6.06 | 3 | nucl |
| 50 | *VcARF16-12* | 26 | VaccDscaff26:17822463..17827109- | 2025 | 674 | 74.38 | 6.03 | 3 | nucl |
| 51 | *VcARF17-1* | 14 | VaccDscaff14:13331772..13340852- | 1725 | 574 | 62.82 | 5.91 | 2 | nucl/chlo/cyto |
| 52 | *VcARF17-2* | 2 | VaccDscaff2:17675083..17684250- | 1722 | 573 | 62.77 | 5.91 | 2 | nucl/chlo/cyto |
| 53 | *VcARF18-1* | 38 | VaccDscaff38:16109186..16113795+ | 2124 | 707 | 78.28 | 6.23 | 14 | nucl |
| 54 | *VcARF18-2* | 6 | VaccDscaff6:26259124..26264169- | 2124 | 707 | 78.34 | 6.23 | 14 | nucl |
| 55 | *VcARF18-3* | 39 | VaccDscaff39:16725386..16730572+ | 2124 | 707 | 78.31 | 6.23 | 14 | nucl |
| 56 | *VcARF18-4* | 37 | VaccDscaff37:14535659..14540819- | 2124 | 707 | 78.31 | 6.23 | 14 | nucl |
| 57 | *VcARF19-1* | 40 | VaccDscaff40:21432199..21439229- | 1743 | 580 | 65.50 | 5.99 | 11 | nucl/plas |
| 58 | *VcARF19-2* | 41 | VaccDscaff41:19942680..19949685- | 1743 | 580 | 65.56 | 6.08 | 11 | nucl/plas |
| 59 | *VcARF19-3* | 40 | VaccDscaff40:21377071..21385465- | 2916 | 972 | 107.96 | 5.98 | 10 | NA |
| 60 | *VcARF19-4* | 12 | VaccDscaff12:31908720..31918385- | 3222 | 1073 | 118.64 | 6.08 | 14 | nucl |

nucl, nucleus; chlo, chloroplast; cyto, cytoplasm; plas, plasm; vacu, vacuole; mito, mitochondria
