# Supplementary material for: Identification of ARF family in blueberry and its potential involvement of fruit development and pH stress response
Source: BMC Genomics. 2022 Apr 27;23:329. doi: 10.1186/s12864-022-08556-y (PMC9047364; doi:10.1186/s12864-022-08556-y)
Supplement: Supplementary file 4 — Additional file 4: Figure S1. Phylogenetic analysis of ARF genes in blueberry and Arabidopsis. The CDS sequences of AtARFs were downloaded from the TAIR website (www.arabidopsis.org). A maximum likelihood tree was generated with the CDS sequences of the ARF genes using the MEGA7 software. [file 12864_2022_8556_MOESM4_ESM.pdf]

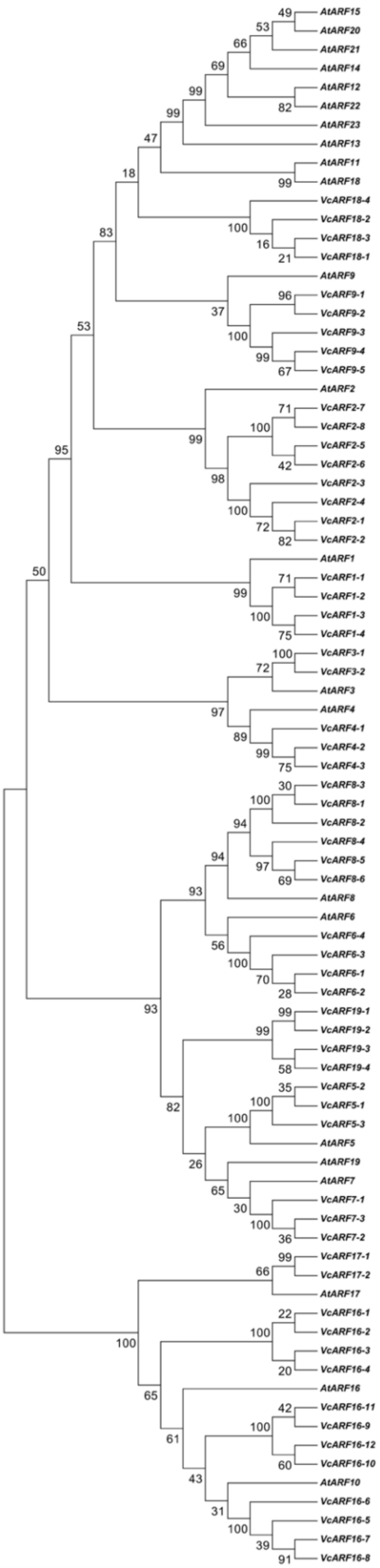
AtARF15
AtARF20
AtARF21
AtARF14
AtARF12
AtARF22
AtARF23
AtARF13
AtARF11
AtARF18
VcARF18-4
VcARF18-2
VcARF18-3
VcARF18-1
AtARF9
VcARF9-1
VcARF9-2
VcARF9-3
VcARF9-4
VcARF9-5
AtARF2
VcARF2-7
VcARF2-8
VcARF2-5
VcARF2-6
VcARF2-3
VcARF2-4
VcARF2-1
VcARF2-2
AtARF1
VcARF1-1
VcARF1-2
VcARF1-3
VcARF1-4
VcARF3-1
VcARF3-2
AtARF3
AtARF4
VcARF4-1
VcARF4-2
VcARF4-3
VcARF8-3
VcARF8-1
VcARF8-2
VcARF8-4
VcARF8-5
VcARF8-6
AtARF8
AtARF6
VcARF6-4
VcARF6-3
VcARF6-1
VcARF6-2
VcARF19-1
VcARF19-2
VcARF19-3
VcARF19-4
VcARF5-2
VcARF5-1
VcARF5-3
AtARF5
AtARF19
AtARF7
VcARF7-1
VcARF7-3
VcARF7-2
VcARF17-1
VcARF17-2
AtARF17
VcARF16-1
VcARF16-2
VcARF16-3
VcARF16-4
AtARF16
VcARF16-11
VcARF16-9
VcARF16-12
VcARF16-10
AtARF10
VcARF16-6
VcARF16-5
VcARF16-7
VcARF16-8

**Figure S1**. Phylogenetic analysis of *AR* *F* genes in blueberry and Arabidopsis. The CDS sequences of *AtARF*s were downloaded from the TAIR website (www.arabidopsis.org). A maximum likelihood tree was generated with the CDS sequences of the *ARF* genes using the MEGA7 software.
