# Supplementary material for: Identification of ARF family in blueberry and its potential involvement of fruit development and pH stress response
Source: BMC Genomics. 2022 Apr 27;23:329. doi: 10.1186/s12864-022-08556-y (PMC9047364; doi:10.1186/s12864-022-08556-y)
Supplement: Supplementary file 5 — Additional file 5: Figure S2. Transcript profiling of the potential targets or downstream genes of VcARFs. (A) Transcript profiling during fruit development (five stages: pad, cup, mg, pink, ripe). (B-C) Transcript profiling in response to different pH conditions (pH4.5 and pH6.5) in pH-sensitive Vaccinium corymbosum (B) and pH-tolerant Vaccinium arboretum (C). The color scale beside the heat map indicates gene expression levels, low transcript abundance indicated by blue color and high transcript abundance indicated by red color. The heatmaps were generated using the software TBtools (Version 1.098689, https://github.com/CJ-Chen/TBtools/releases). [file 12864_2022_8556_MOESM5_ESM.pdf]

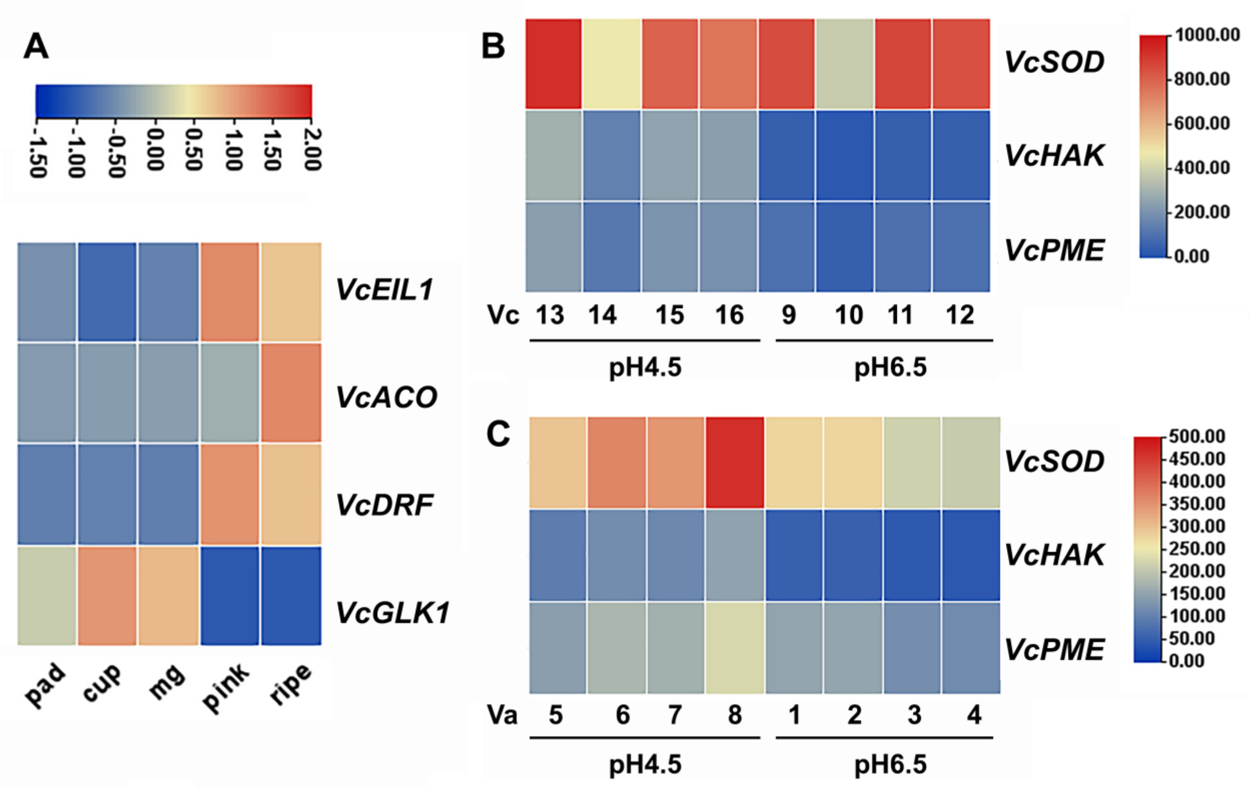

Figure S2 Transcript profiling of the potential targets or downstream genes of VcARFs. (A) Transcript profiling during fruit development (five stages: pad, cup, mg, pink, ripe). (B-C) Transcript profiling in response to different pH conditions (pH4.5 and pH6.5) in pH-sensitive *Vaccinium corymbosum* (B) and pH-tolerant *Vaccinium arboretum* (C). The color scale beside the heat map indicates gene expression levels, low transcript abundance indicated by blue color and high transcript abundance indicated by red color. The heatmaps were generated using the software TBtools (Version 1.098689, https://github.com/CJ-Chen/TBtools/releases).
